# Phytoremediation for the indoor environment: a state-of-the-art review

**DOI:** 10.1007/s11157-023-09644-5

**Published:** 2023-02-27

**Authors:** S. Matheson, R. Fleck, P. J. Irga, F. R. Torpy

**Affiliations:** 1 Plants and Environmental Quality Research Group, Faculty of Science, School of Life Sciences, University of Technology Sydney, Broadway, NSW 2007 Australia; 2 Plants and Environmental Quality Research Group, Faculty of Engineering and Information Technology, School of Civil and Environmental Engineering, University of Technology Sydney, Sydney, Australia

**Keywords:** Sustainable infrastructure, Sustainable development goals, Green walls, Biofilter, Indoor air quality, Green building

## Abstract

Poor indoor air quality has become of particular concern within the built environment due to the time people spend indoors, and the associated health burden. Volatile organic compounds (VOCs) off-gassing from synthetic materials, nitrogen dioxide and harmful outdoor VOCs such benzene, toluene, ethyl-benzene and xylene penetrate into the indoor environment through ventilation and are the main contributors to poor indoor air quality with health effects. A considerable body of literature over the last four decades has demonstrate the removal of gaseous contaminants through phytoremediation, a technology that relies on plant material and technologies to remediate contaminated air streams. In this review we present a state-of-the-art on indoor phytoremediation over the last decade. Here we present a review of 38 research articles on both active and passive phytoremediation, and describe the specific chemical removal efficiency of different systems. The literature clearly indicates the efficacy of these systems for the removal of gaseous contaminants in the indoor environment, however it is evident that the application of phytoremediation technologies for research purposes *in-situ* is currently significantly under studied. In addition, it is common for research studies to assess the removal of single chemical species under controlled conditions, with little relevancy to real-world settings easily concluded. The authors therefore recommend that future phytoremediation research be conducted both *in-situ* and on chemical sources of a mixed nature, such as those experienced in the urban environment like petroleum vapour, vehicle emissions, and mixed synthetic furnishings off-gassing. The assessment of these systems both in static chambers for their theoretical performance, and *in-situ* for these mixed chemical sources is essential for the progression of this research field and the widespread adoption of this technology.

## Introduction

### Growing concern of air pollution

Currently, urban air pollution is a significant health risk for urban dwellers worldwide, accounting for 5% of the global disease burden (Cohen et al. 2017), and this is projected to increase with the rising urban population. In 2015 the United Nations proposed the “Sustainable Development Goals” (SDGs) to the 193-member nations, officially known as the “2030 Agenda for Sustainable Development” (United Nations General Assembly, 2015). In 2018 55% of the global population lived in urban centres, and it has been estimated that this will increase to 68% by 2050 (Desa 2019). Poor air quality is such an issue that it is directly addressed within the SDG targets: SDG 3.9 (substantial reduction of health impacts from hazardous chemicals and air, water, and soil pollution) and SDG 11.6 (reduction of adverse per capita environmental impacts of cities, including paying special attention to air quality and other waste management).

Historically there have been several events that have drawn the attention of governments worldwide (Boyd 1960), to establish air quality assessment systems, such as the Los Angeles photochemical smog in the 1940s (Kuwata 1974) and the London smog in 1952 (Logan 1953). Consequently, the World Health Organisation (WHO) and other environmental agencies have established guidelines for air pollution for a range of contaminants (Table 1). Despite the development of air quality guidelines and monitoring efforts, significant health implications and mortality due to air pollution remain an issue for much of the world (Brunekreef and Holgate 2002; Organization 2006; Pope III and Dockery 2006).

**Table 1:** Air Quality Indices and guidelines for some developed and developing countries (Australia 2019; Britain 2020; Li et al. 2018; Yang et al. 2019)

Country/ Area	National Guideline	Year of implementation	Major pollutants	Measuring Time (Hour average)	Safe limits
World Health Organisation	Air Quality Guidelines (AQG)	2021	PM_2.5_	24	15 µg m^−3^
			O_3_	8	100 µg m^−3^
			NO_2_	24	25 µg m^−3^
			SO_2_	24	40 µg m^−3^
			CO	24	4 µg m^−3^
United States	Air Quality Index (AQI)	2016	O_3_	8	0.1 ppm
			PM_2.5_	24	15 ppm
			CO	8	50 ppm
			SO_2_	1	5 ppm
			NO_2_	1	5 ppm
Australia	National and Environmental Protection Measure for Ambient Air Quality (Air NEPM)	2016	CO	8	30 ppm
			Lead	1	20 µg/dL
			NO_2_	1	3 ppm
			PM_2.5_	24	0.1 mg/m^3^
			SO_2_	4	2 ppm
The United Kingdom	Daily Air Quality Index (DAQI)	2012	O_3_	8	0.1 ppm
			NO_2_	1	0.5 ppm
			SO_2_	0.25	0.5 ppm
			PM_2.5_	24	10 mg.m^3^
People's Republic of China	Air Quality Index (AQI)	2012	SO_2_	1	150 µg m^−3^
			NO_2_	24	120 µg m^−3^
			PM_2.5_	24	150 µg m^−3^
			CO	1	3.5 ppm
			O_3_	8	160 µg m^−3^
			PM_10_	24	
European Union	Air pollution Index (API)	2007	PM_2.5_	24	50 µg m^−3^
			SO_2_	24	125 µg m^−3^
			O_3_	8	120 µg m^−3^
			NO_2_	1	200 µg m^−3^
			CO	8	20 ppm

This table focuses on a limited range of criteria pollutants, and does not cover the full range of domestic guidelines

In urban centres, vehicle emissions are the primary source of harmful pollutants. Combustion reactions emit a complex mixture of suspended particles (PM; Particulate Matter) and gaseous pollutants including nitrogen dioxide (NO_2_) and ozone (O_3_), which make up the majority of harmful pollutants (Joshi 2008; Wang et al. 2019). In developed and many developing countries, SO_2_ and Pb pollution has become less of an issue than the past due to tighter industrial regulation and implementation of unleaded fuels; this has seen reduction in SO_2_ emissions across Europe by more than 60% between 1990 and 2004 (Vestreng et al. 2007) (Table 2).

**Table 2:** Occupational exposure limits (OELs) of a cohort of administrations for prominent VOCs

Administration	Chemical name	TWA (ppm)	STEL (ppm)
Safe Work Australia (SFA) (Australia 2019)	Benzene Toluene Ethyl benzene Xylene Formaldehyde	1 50 100 80 1	Undisclosed 150 125 150 2
Occupational Health and Safety administration (OSHA, United States of America) (Kostoff 2018)	Benzene Toluene Ethyl benzene Xylene Formaldehyde	1 200 100 300 2	5 500 150 150 0.75
Heath Safety Executive (HSE, United Kingdom) (Britain 2020)	Benzene Toluene Ethyl benzene Xylene Formaldehyde	1 50 100 50 2	Undisclosed 100 125 100 2
European Chemical Agency (European Union) (Commission 2000)	Benzene Toluene Ethyl benzene Xylene Formaldehyde	1 50 100 50 0.3	Undisclosed 100 200 100 0.6
Ministry of Labour & Employment, Government of India (Government of India 1948)	Benzene Toluene Ethyl benzene Xylene Formaldehyde	0.5 100 Undisclosed 100 1	2.5 150 Undisclosed 150 2

Exposure limits for the time weighted average (over 8 h) and the short-term exposure limit (15 min) are presented for Australia, USA, UK, EU and India

Most countries have taken measures to reduce air pollution, with policies and measures such as vehicle emission controls and the use of unleaded fuels implemented, however, high traffic densities and industrial emissions within cities regularly lead to urban pollution concentrations exceeding the World Health Organisation (WHO) guidelines outlined in Table 1 (Hoek et al. 2013). Urban infrastructures such as car parks, traffic tunnels, and underpasses trap gaseous pollutants at ground level, preventing dispersion into the atmosphere and creating pocket regions with high concentrations of pollution (Abhijith et al. 2017; Venkatram and Schulte 2018). These high pollutant scenarios are usually within areas with high population densities, resulting in consistent inhalation of pollutants by people who live and work in these areas. Long-term exposure to these pollutants is associated with lower pulmonary function and cancer, with increases in pollution greater than 10 μg/m^3^ of PM correlated with increases in all-cause mortality and hospital emissions (Gryparis et al. 2004; Hoek et al. 2013; Jindal 2007).

### Air quality within indoor environments

In an effort to reduce building heating, ventilation and air conditioning (HVAC) energy consumption, buildings have become increasingly sealed from the ambient environment. With an ever-increasing urban population, where people spending on average 90% of their time indoors (Zhang 2004), cities have developed a reliance on mechanical ventilation (Yu and Kim 2010) for air conditioning and purification. Conventional mechanical technologies such as air filters with a minimum efficiency reporting value (MERV) rating of 8–13 are used within HVAC units to filter the air. However, these systems are only effective for removal of PM, and are incapable of gaseous pollutant filtration (Chen et al. 2005). Globally, the range of pollutants to which indoor occupants are exposed are largely dependent on building ventilation type, geographical location and socioeconomic development (Colbeck et al. 2010). Without the capability to remove gaseous pollutants and the highly sealed interior of buildings, it is common for indoor environments to be 3–5 times more polluted than ambient outdoor air (Jafari et al. 2015). As such, the accumulation of indoor pollutants can often lead to negative health effects ranging from those as simple as discomfort and loss of productivity to acute health effects (Challoner and Gill 2014; Ghaffarianhoseini et al. 2018). Thus, there is a need for air cleaning technologies that are both energy efficient and capable of removing of the key gaseous pollutants.

Methods are increasingly employed to control indoor air pollution including eliminating the pollutant at the source through altering the building structure i.e. building external walls (Irga et al. 2019; Kaunelienė et al. 2016; Moya et al. 2019), optimizing ventilation and modifying individuals’ behaviour by altering cooking methods and reducing exposure to smoke (Rounaghi and Eshghi 2017). Various systems can also be used individually or in combination for the removal of organic gaseous pollutants from contaminated air within buildings such as ventilation, ozonation, ultraviolet (UV) photolysis, cold-plasma or non-thermal plasma (NTP), air stripping and membrane separation (Guieysse et al. 2008; Jimenez-Relinque and Castellote 2014). However, these processes require large amounts of energy and capital investment (Sriprapat et al. 2014a, b). Biofiltration by botanical systems represents an alternative method to treating indoor air. These systems require lower energy and capital investment, making them a more sustainable and practical method in many applications (Agarwal et al. 2019; Brilli et al. 2018; Cummings and Waring 2020; Han and Ruan 2020).

In developed nations the most prominent pollutants are VOCs (outlined in Table 2), and PM, however, the majority of literature focuses on a select range of specific VOCs and their effects (de Gennaro et al. 2015; Kim et al. 2010). In reality occupants are exposed to a variety of harmful volatiles simultaneously, with the main sources of these being combustion emissions followed by petroleum vapour (Al-Harbi et al. 2020; Brauer et al. 2016; Wolkoff 2013).

#### Ambient VOCs

The BTEX group are known carcinogens that are frequently detected in ambient air, especially in areas with high traffic emissions, industrial and commercial activity as well as petroleum evaporation from vehicles such as in indoor garages and fuel stations (Leong et al. 2002). BTEX are considered major air pollutants due to their hazardous properties and propensity to readily volatilise and distribute over large areas (Adams et al. 2001; Durmusoglu et al. 2010). Exposure limits to BTEX is regulated by administrations such as the Occupational Safety and Health administration (OSHA) and Safe Work Australia (SWA), which use short-term exposure limits (STEL) defining maximum concentrations workers can be exposed to in a 10–15 min timeframe as well as a time weighted average (TWA) for exposure standards over a 8-h work day (Table 2) (Davidson et al. 2021). However, these only define safety limits for exposure to individual solvents, whereas in an occupational setting workers are likely to be exposed to multiple BTEX chemicals simultaneously (Davidson et al. 2021). Exposure to these carcinogenic aromatic species has been associated with increased pulmonary pathology, nasopharyngeal and laryngeal cancer, cataract and lung cancer (Adams et al. 2001; Davidson et al. 2021; Durmusoglu et al. 2010; Godoi et al. 2013; Leong et al. 2002).

#### Indoor volatile organic compounds (VOCs)

In addition to outdoor VOCs, a diverse range of contaminants are emitted via off gassing from structural building materials such as furnishings, adhesives, floor and wall coverings, cleaning products and plastics (Yu and Kim 2010). Environmental factors such as temperature and humidity are also known to impact indoor VOC concentrations (Gunschera et al. 2013; Zhang et al. 2007). A monitoring campaign conducted by Zhang et al. (2020) found positive correlations of temperature and humidity on formaldehyde concentrations within households and public places in Harbin, China. With multiple indoor sources and a sealing of the building envelope to reduce energy consumption within many commercial spaces, has led to the persistence of indoor VOCs and increased exposure for building occupants leading to a phenomenon known as “sick building syndrome” (Jia et al. 2008; Joshi 2008). Short-term exposure to indoor-generated VOCs can cause fatigue, headaches, dizziness, nausea, lethargy and depression (Policy and Analysis 1987), and chronic respiratory effects and lung cancer have been associated with long-term exposure (Hodgson et al. 1991). A study by Pappas et al. (2000) notes that indoor TVOC concentrations in the range of 25–50 mg/m^3^ with a 4-h exposure may lead to detectable negative upper and lower respiratory function. While these concentrations represent what might be found in very poor quality indoor air, they are still lower than those typically found in industrial settings (Mølhave 1982; Mølhave and Møller 1979).

#### Conventional indoor air quality management

HVAC systems, alongside their temperature and humidity functions, are commonly used within the developed world to replace polluted indoor air with outdoor air through their ventilation function (Lin and Chen 2014; Wargocki et al. 1999). This is considered the most effective and simplest method for indoor air quality management (Torpy et al. 2015), however, due to the need for HVAC to manage temperature differentials between outdoor and indoor air, a significant amount of electrical energy is required in climates where the ambient temperature is either hotter or colder than the required indoor temperature. This energy use, particularly in industrialised nations, is substantial, with ~ 26.5% of all energy use in the US being attributed to HVAC (Ben-David and Waring 2016). Nearly all mechanically ventilated buildings use filters to prevent some inlet air PM infiltrating from outdoors (Ben-David and Waring 2016; Quang et al. 2013). Nevertheless, common HVAC systems can only filter a proportion of PM from influent air, which results in indoor PM concentrations maintaining a correlation with proximal outdoor concentrations (Guo et al. 2010; Morawska et al. 2013; Morawska and Clark 2000). Common HVAC PM filters with a minimum efficiency reporting value (MERV) of 4, 6, 10 and 11 report removal efficiencies of less than 20% across all particle sizes (Stephens and Siegel 2013). More efficient filters are available, however increased PM filtration efficiencies comes with higher maintenance, greater energy usage, reduced sustainability and remains incapable of filtering gaseous pollutants (Montgomery et al. 2012; Quang et al. 2013). Naturally ventilated buildings generally use less energy than mechanically ventilated buildings, however they have the potential to provide greater exposure to PM from outdoor sources (Ben-David and Waring 2016). Also, due to outdoor climatic conditions, natural ventilation is not always possible (Guieysse et al. 2008).

As a result of the increasing air-tightness of buildings and resultant accumulation of indoor sourced pollutants there is an increased need to purify the air inside buildings (Guieysse et al. 2008). Air purification methods such as ionization, activated carbon absorption, ozonation and photocatalysis may be integrated into a building’s ventilation system (Chen et al. 2005; Guieysse et al. 2008; Luengas et al. 2015). These techniques are efficient at removing singular pollutant types, however typical indoor environments have numerous pollutants with diverse physio-chemical properties making effective joint treatment difficult (Luengas et al. 2015). Mechanical methods are also all expensive, potentially hazardous (ozonation), and require high energy usage. The development of air-cleaning technologies which are energy efficient and capable of treating a wide range of air pollutants is crucial for the reduction of health impacts caused by hazardous air pollution (SDG 3.9), and will help reduce its adverse environmental impacts on cities (SDG 11.9).

## Plants as a phytoremediation technology

### Passive and active potted plants

**Fig. 1:**
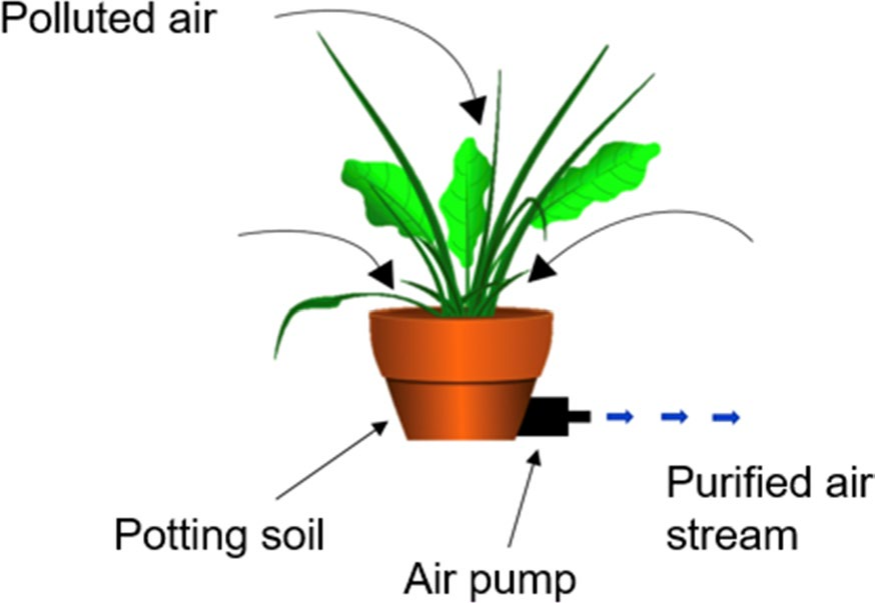
Wolverton et al. (1984) active biofilter design (image adapted by author)

Botanical biofiltration of VOCs was proposed as a method for the purification of indoor spaces by NASA scientists (Wolverton and McDonald 1982) while investigating potted-plants for their innate ability to phytoremediate toxic compounds. While passive plant systems significantly reduced ambient VOC concentrations in the model spacecraft that were used by NASA, the remediation potential of static plant systems is rate limited by the diffusion rates of pollutants from their sources to the plant foliage and substrate (Wolverton and McDonald 1982) which prevents the control of their decontamination efficiency (Khalifa et al. 2022). Therefore Wolverton et al. (1984) proposed the adoption of active airflow for an increase in phytoremediation potential (Fig. 1). This idea was further developed by Darlington et al. (2001) with the release of his commercial active green walls, and later built upon by other researchers with the introduction of active airflow and substrate development for rhizospheric remediation of various VOCs (Darlington et al. 2001; Pettit et al. 2018a).

The primary removal mechanism of VOCs by active botanical biofilters is the delivery of a contaminated airstream to the rhizospheric bacteria hosted by the plant through the use of mechanised components (Deng and Deng 2018; Mannan and Al-Ghamdi 2021). This allows for an increase in the delivery rate of pollutants to the rhizosphere, which is largely believed to drive the phytoremediation process termed rhizodegradation (Torpy et al. 2015). Compounds released from plants, such as sugars, amino acids, or enzymes, can stimulate bacterial growth in the soil and reversely stimulate microbial degradation of delivered pollutants by releasing exudates/enzymes into the rhizosphere (Lee et al. 2021; Ma et al. 2016).

### *Removal of VOCs *via* passive potted plant systems*

**Fig. 2:**
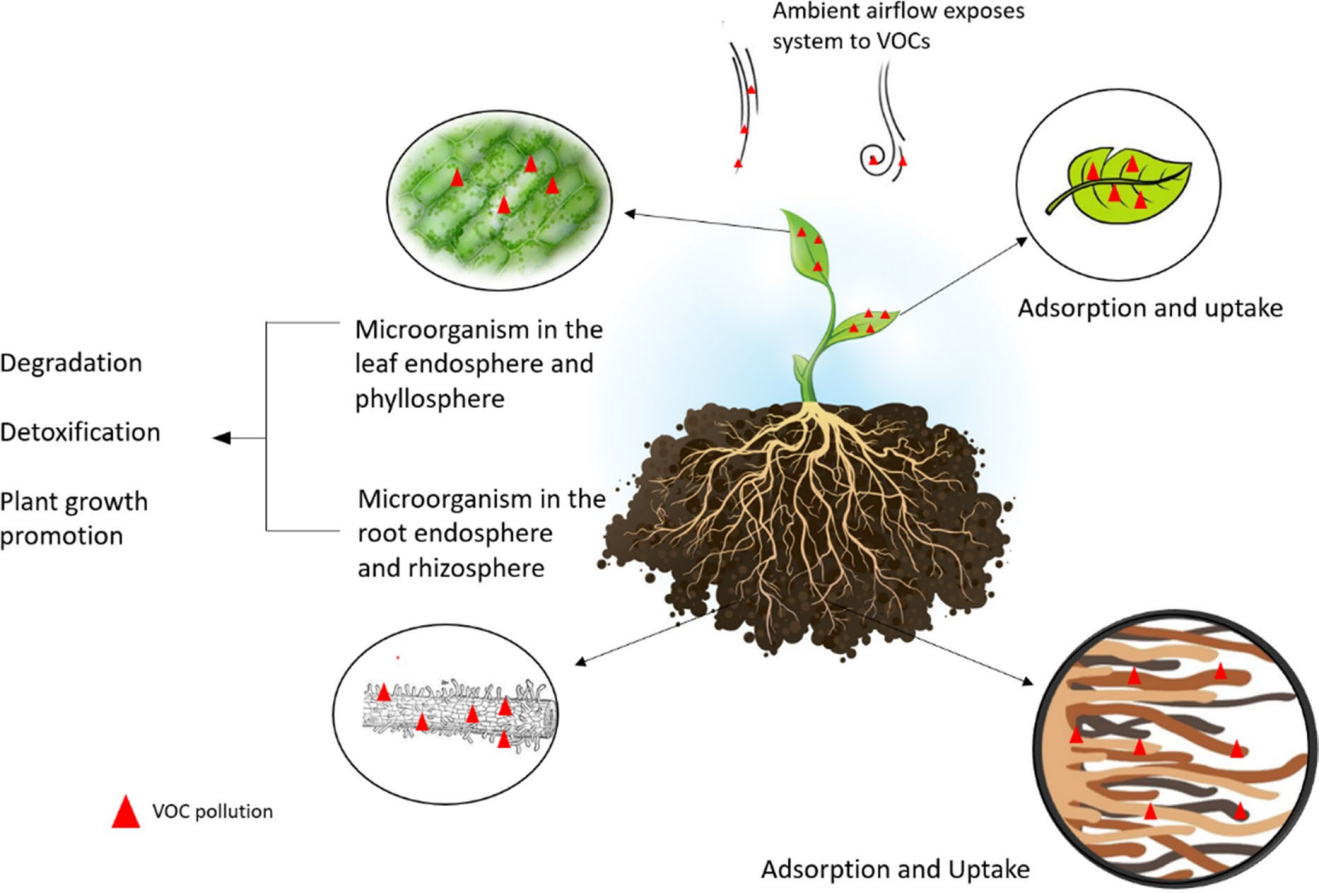
Foliar and rhizospheric removal mechanisms for the phytoremediation of VOCs by passive plant systems (image by Author)

Plant systems are able to remediate air contaminants by three different routes: removal through aerial parts of the plant and phyllospheric organisms (Wei et al. 2017), removal by soil microorganisms (rhizosphere) and removal by the growing media (Aydogan and Montoya 2011) (Fig. 2). Since the initial experiments conducted by Wolverton et al. (1982, 1984), numerous laboratory chamber test studies of both passive and active potted systems have demonstrated the potential for significant improvement in indoor air quality (IAQ) (Table 3). The pollutants most commonly tested are chemicals from the BTEX group, as well as formaldehyde, due to them being the most hazardous VOC pollutants. Studies vary in respect to growth substrate composition, concentration of VOC, and plant selection. Experiments generally follow the same methodology, consisting of placing one or more potted plants in a sealed chamber, having VOCs introduced and drawdown being measured over time to determine the pollutant removal rate.

**Table 3:** A summary of passive pot plant studies (2012–2022) that detail the removal of various VOCs from static chambers, for studies before this time period see (Pettit et al. 2018b)

Study	Year	Pollutants	Starting concentrations	Plant species	Substrate information	Chamber volume	Removal rate/efficiency	Removal mechanism
(Treesubsuntorn and Thiravetyan)	2012	Benzene	20 ppm	*C. seifrizii,* *S. aureus,* *S. trifasciata,* *P. domesticum,* *I. craib,* *M. acuminate* *E. aureum,* *and D. sanderiana*	Pot covered with aluminium foil	N/A	Removal at 72 h range from 43–77% depending on species	Benzene can be removed through both stomatal uptake and through crude wax, during dark conditions cuticle wax uptake was more prevalent However, light conditions still revealed optimum pollutant uptake
(Irga et al.)	2013	Benzene	25 ppmv	*S. podophyllum*	Planted Hydroculture Substrate only	15.86 L 15 L 500 ml	50% removal at 1444 µg/m^3^/h^1^/pot 50% removal at 739 µg/m^3^/h^1^/pot 50% removal at 519 µg/m^3^/h^1^/pot	Benzene removal within hydroculture substrate was slower than traditional potted plants. Concluded that the more diverse bacterial community within the potting substrate increased VOC removal
(Sriprapat and Thiravetyan)	2013	Benzene, Toluene, Ethylbenzene, Xylene	20 ppm of each BTEX	*Z. zamiifolia*	1:1 soil to coconut coir	15.6 L	0.96 ± 0.01 (B), 0.92 ± 0.02 (T), 0.92 ± 0.02 (E), 0.86 ± 0.07 (X), mmolm^−2^ at 72 h	Benzene may be taken up faster than other BTEX due being a smaller molecule. BTEX toxicity was not found during 3-day fumigation The ratio of stomata and cuticles showed that 80% of benzene, 76% of toluene, 75% of ethylbenzene, and 73% of xylene were removed by stomatal pathways, while 20, 23, 25, and 26% were removed by non-stomatal pathways or cuticles
(Torpy et al.)	2013	Benzene	25 ppmv (80 mg/m^3^)	*S. wallisi*	Standard potting	216 L	Bio stimulation increased removal rates by ~ 27%	Provided evidence of the importance of microorganisms in pollutant removal, bio stimulated plants demonstrated higher benzene removal rates
(Treesubsuntorn et al.)	2013	Benzene	20 ppm	21 ornamental plants from commercial Thai shop	No pot just leaf	6 L	1.10 – 23.46 µmol/g of plant material over 3 days	High quantities of wax in the cuticle produced higher removal rates for benzene across the plant species
(Kim et al.)	2014	Toluene, Xylene	1 µ/L	*F. japonica and D. fragrans*	5:1:1 (Bark, humus, sand)	996.3 L	N/A	*F. japonica* exhibited a more rapid rate of removal for toluene and xylene than *D. fragrans* Efficiency of VOC removal increased as the root zone volume increased
(Sriprapat, Suksabye, et al.)	2014	Toluene, Ethylbenzene	20 ppm or 12 µm	12 ornamental plant species from Thailand florest	1:1 soil to coconut coir	15.6 L	~ 77% removal at 72 h (Toluene) across 12 plants ~ 70% removal at 72 h (Ethylbenzene) across 12 plant	Highest toluene and ethylbenzene removal were observed in *S. trifasciata* and *C. comosum* respectively Cuticle wax composition showed higher removal. Hexadecenoic acid was present
(Mosaddegh et al.)	2014	Benzene, Toluene, Ethylbenzene, Xylene, Methanol, Acetone, Acetonitrile	2 ppm	*D. deremensis* and *O. microdasy*	Soil	50 L	3.2 mg/m^3^ per day (*O.microdasy*) 1.46 mg/m^3^ per day (*D.deremensis*)	Benzene removal pathways by plant or substrate media was not explored
(Su and Liang)	2015	Formaldehyde	30, 60 or 120 mg/L	*C. comosum*	Hydroponically with Hoagland’s solution	52.5 L	135 µg/h/plant (maximum)	Majority of formaldehyde was taken up into the plant’s roots Plant leaves showed an ability to dissipate formaldehyde which increased over time
(Kim et al.)	2016	Toluene, xylene	0.5 µL/L of toluene with 0.3 µL/L of xylene	*S. actinophylla and F. benghalensis*	5:1:1 (Bark, humus, sand)	996.3 L	Removal efficiency of toluene and xylene was 13.3 and 7.0 µg/m^3^/m^2^ leaf area over a 24 h period in *S. actinophylla*, and was 13.0 and 7.3 µg/m^3^/m^2^ leaf area for *F. benghalensis*	Concluded that root zone is the main contributor for toluene and xylene removal with transport to the plant stem also playing a role, with 47% of toluene and 60% of xylene transported via plant stem for both species
(Sriprapat and Thiravetyan)	2016	Benzene	170 µg	*S. podophyllum,* *S.trifasciata,* *E.milii,* *C.comosum,* *E.aureum,* *D.sanderiana,* *H.helix, and* *C. ternatea*	Murashige and Skoog (MS) medium supplemented with Gamborg vitamin	undisclosed	25.3 – 34 µmol m^−2^ h^−1^	Most efficient plant for benzene removal was *C. comosum* *Cronobacter* sp*., Pseudomonas* sp*.* and* Enterobacter* sp. Highlighted importance of endophytic and epiphytic bacteria in benzene removal
(Hörmann et al.)	2017	Toluene, 2 – ethylhexanol	20.0 mg/m^3^ (Toluene) and 14.6 mg/m^3^ (2-ethylhexanol)	*D. maculata and S. wallisii*	Potting soil	240 L	~ 70% (Toluene) 48 h ~ 90% (2-ethylhexanol)	No significant difference between empty chambers and planted chambers for 2-ethylhexanol removal Significant VOC adsorption by both chamber surfaces and aerial plant parts and potting soil was evident for toluene
(Chen et al.)	2017	Formaldehyde	≥ 5 ppm	*H. helix*	Sterilized media	225 L	~ 4 ppm over 17.1 h	Showed that potted *H. helix* reduced 70% of the required time to reach 0.5 ppm of gaseous formaldehyde when compared with natural dissipation. Potted *H. helix* also removed residual formaldehyde
(Setsungnern et al.)	2017	Benzene	500 ppm	*C.comosum*	Roots wrapped in tissue paper and aluminium foil	15.6 L	31.37% removal under 1:1 LED light 24.75% removal under fluorescent light	Benzene removal by plants was best under LED light, helping plants produce more brassinosteroids to degrade benzene and utilize it as a carbon source
(Hörmann et al.)	2018	Toluene, 2-ethylhexanol	20.0 mg/m^3^ (Toluene) and 14.6 mg/m^3^ (2-ethylhexanol)	*D. maculata, S. wallisii* and *A. densiflorus*	*Undisclosed*	240 L	1.4 – 1.5 L h^−1^ m^−2^	Specifically looked at aerial plant part removal rather than the whole system. Concluded aerial plant parts have no major impact on chamber air quality
(Teiri et al.)	2018	Formaldehyde	0.66 – 16.4 mg/m^3^	*C. elegans*	Loamy soil	375 L	1.47 mg/m^2^/h	Substantial contribution of soil and roots for formaldehyde removal, attributed to microorganisms
(Budaniya and Rai)	2022	Particulate matter	350 – 750 µg/m^3^	*H.splendens,* *C.macrocarpa, A.heterophylla,* *P.orientalis,* *P.roebelenii,* *E.purpureum,* *D.reflexa,* *S.trifasciata,* *E.aureum,* *F.retusa,* *C. variegatum*	*Undisclosed*	210 L	CADRs; 0.002 ± 0.004 m^3^/h (needle leave plants) 0.084 ± 0.009 m^3^/h (broad-leaved plants	Significantly lower CADRs for passive plant systems compared to filter-based purifiers (170–800 m^3^/h). Concluded that passive plant systems cannot compete with conventional air purifiers, large quantities of plants would be required to achieve modest indoor PM concentrations
(Liu et al.)	2022	CO_2_, HCHO, TVOC, PM_10_, PM_2.5_	795 ppm (CO_2_) 120 µg m^−3^ (HCHO) 2,786 µg m^−3^ (TVOC) 87	*E.aureum*	Potting soil	216 L	Removal efficiency over 12 h; 26.87% (CO_2_) 61.73% (HCHO) 30.04% (TVOC) 81.97% (PM_10_) 79.2% (PM_2.5_)	Removal pathways were not explored

Table includes pollutant type, starting concentration, plant species, substrate information, chamber volume and efficiency. Removal mechanisms as described by the authors of each study are also presented

Static chamber experiments have provided ‘proof of concept’ for the use of plants as biofiltration devices, where numerous studies have demonstrated microbial degradation to be the main proprietor for VOC removal (Aydogan and Montoya 2011; Hörmann et al. 2018; Irga et al. 2013; Kim et al. 2010, 2014; Orwell et al. 2004; Sriprapat and Thiravetyan 2013; Su and Liang 2015; Teiri et al. 2018; Torpy et al. 2013; Wood et al. 2006, 2002). While the contribution of the aerial plant parts is significantly smaller when compared to the rhizosphere, several studies have shown that plant foliage is able to remove some gaseous VOCs (Sriprapat et al. 2014a, b; Tani and Hewitt 2009; Treesubsuntorn et al. 2013) to a measurable degree. These studies identified the affinity for some VOCs to diffuse across the cuticle of the plant, suggesting that removal is dependent on the quantity of wax and the chemical structure of the epicuticle (Treesubsuntorn et al. 2013). Aydogan and Montoya (2011) found when removing formaldehyde, both the root zone and aerial plant parts were capable of VOC removal independently, however, removal by the root zone occurred at a significantly faster rate. Contrastingly, Hörmann et al. (2017) observed similar VOC removal rates for the plant foliage when covering the substrate. Interestingly, Hörmann et al.’s (2017) findings may be explained by the works of Su and Liang (2013, 2015) where they observed the primary mechanism of the plant foliage was to deliver VOCs to the rhizosphere through the plants vascular system via the phloem (Gupta et al. 2017). Inhabited microbes on the leaf surfaces work to detoxify part of the adsorbed or absorbed pollutant through degradation, sequestration or transformation, remaining pollutants are then transferred to the soil where rhizospheric organisms further detoxify them via the microbial metabolism pathway (Prigioniero et al. 2021; Teiri et al. 2021).

While the scientific consensus is that the rhizosphere is the main proprietor for VOC removal (Torpy et al. 2013), the entire potted ecosystem is required for effective VOC removal, with the symbiotic relationship governed by the plant providing the structure and chemical signalling for the rhizospheric bacteria (Hirsch and Fujishige 2012; Kim et al. 2014; Shao et al. 2020; Wood et al. 2002; Xu et al. 2011). As the phytoremediation potential of these systems is biologically driven, several studies have observed a notable increase VOC removal efficiency with repeat exposure to a single VOC due to specific biostimulation of the bioremediation-active rhizospheric microbial community (Orwell et al. 2004; Torpy et al. 2013; Wood et al. 2006). Varying results for multiple VOC removal and plant interactions have been exhibited. Sriprapat and Thiravetyan (2013) recorded higher benzene removal in plants over the other BTEX VOCs, relating this to benzene’s smaller molecular size allowing faster uptake. One specific example of selective VOC removal was highlighted by the work of Orwell et al. (2006) where the simultaneous degradation of toluene and *m*-xylene was observed to increase after previous benzene removal by plants. Orwell et al. (2006) suggested that this was a result of the saturation of the catechol,1,2,dioxygenase enzyme (the catechol ring-splitting step in the microbial degradation of benzene and toluene). This is in correspondence with findings from Yeom et al. (1997) who observed the need for toluene presence to sustain *m*-xylene removal rates.

Numerous studies have also noted the ability of a plant’s growth substrate to adsorb VOCs. Substrates of different compositions have been trailed for their capacity to influence VOC removal (Hörmann et al. 2017; Wang et al. 2012). Irga et al. (2013) observed a difference in removal efficiency for benzene between potted-plants grown in potting mix and in hydroculture. The authors concluded that the density and diversity of the microbial community within the substrate was a major contributing factor for benzene removal efficiency. Aydogan and Montoya (2011) provided further evidence of the role substrate plays in VOC removal, with their work showing higher removal rates for formaldehyde with the incorporation of activated carbon over expanded clay and grow stone substrates, attributing this to both the high adsorption capacity of activated carbon and its affinity to provide sufficient microbial sites that could lead to increased VOC removal. The VOC removal mechanisms of potted plant systems have been further explored within previous reviews (Irga et al. 2018; Pettit et al. 2018b). Currently, the majority of chamber experiments have assessed the removal of single VOCs, such as benzene, toluene, hexane, xylene and formaldehyde (Baosheng et al. 2009; Cornejo et al. 1999; Porter 1994; Wood et al. 2002), whereas indoor occupants may be exposed to air containing hundreds of VOCs (Joshi 2008; Meciarova and Vilcekova 2016). The ability of botanical biofilters to remove an azeotropic mixture of VOCs has historically remained unexplored until recently (Morgan et al. 2022). Additionally, the exact mechanisms of removal *in-situ* for a range of physiochemically and behaviourally different VOCs is difficult to determine due to the lower concentrations seen *in-situ,* compared to the elevated levels that have typically been used in the previously mentioned research. This is an area of research requiring future effort.

While the vast number of static chamber trials have provided knowledge regarding the efficacy of potted plants to remove VOCs, generalising their results to real *in-situ* indoor air concentrations within larger rooms is confounded (Budaniya and Rai 2022; Llewellyn and Dixon 2011). Budaniya and Rai (2022) provides results highlighting the inefficiency of potted plants to filter indoor ambient PM, significantly lower removal rates were produced by the passive plant systems tested compared to commercial filter-based air filters. The authors concluded that an unreasonably large quantity of plants would be required to obtain equivalent particle removal rates to conventional filters.

### History of the phytoremediation field

In the formative years of chamber-based experiments, the primary focus of research was exploring the specific removal capacities of VOCs by a wide range of plant species (Kim et al. 2010; Yang et al. 2009; Zhou et al. 2011). While differences between species were observed, the general conclusion was that all plant species were effective at removing VOCs to some degree, and that the driver for phytoremediation was likely to be the rhizospheric microbial community which existed in a symbiotic relationship with the plant (Irga et al. 2013; Torpy et al. 2013). This idea was further affirmed through experimentation where plant parts were isolated using foil or Teflon bags to determine the effect of specific plant parts or the substrate bacterial community (Aydogan and Montoya 2011; Sriprapat et al. 2014a, b; Sriprapat et al. 2014a, b; Treesubsuntorn and Thiravetyan 2012). It was assumed that light intensity would enhance foliar uptake of VOCs through an increase in stomatal conductance (Kondo et al. 1995; Porter 1994), however the results from these experiments were inconsistent, and phytocatalysation may have been a factor in the removal of certain chemical species, especially formaldehyde which is known to be susceptible to photocatalysis (Kondo et al. 1995; Teiri et al. 2018; Xu et al. 2011), whereas more stable aromatics such as benzene and toluene were unaffected (Hörmann et al. 2018; Orwell et al. 2004; Wood et al. 2002). Additionally, it was theorised that the physical and physiochemical properties of a plant’s leaf parts (such as the waxy cuticle) could serve as an adsorption site for some VOCs, however the efficacy of foliage-only phytoremediation is consistently substantially lower than whole-system or rhizosphere only tests (Sriprapat and Thiravetyan 2013). For example, studies where the root zones were successfully isolated have demonstrated higher removal efficiencies than plant-leaf removal only, although the physical removal of the above-ground parts of a plant quickly deteriorate the efficiency of the entire plant-substrate system (Hörmann et al. 2017; Kim et al. 2016; Setsungnern et al. 2017; Treesubsuntorn et al. 2013).

While there is debate amongst some authors on the relative contribution of above or below ground remediation, there is certainly a relationship between the two. Several authors have observed the translocation of VOCs from the plant foliage to the rhizosphere through the phloem, which further indicates the importance of the plant, not only to sustain the rhizospheric community, but to assist in the delivery of pollutants in the absence of active airflow (Aydogan and Montoya 2011; Irga et al. 2013). These findings have led researchers towards biostimulation of the rhizospheric community for the improved removal of VOCs. Increased performance has been achieved through the direct stimulation of the rhizosphere through microbial inoculation, however repeated exposure has also been observed to result in increased performance due to the natural up-regulation of VOC degrading bacteria, by giving them a competitive advantage over non-VOC degrading species (De Kempeneer et al. 2004; Khaksar et al. 2016; Sriprapat and Thiravetyan 2016; Torpy et al. 2013). While this body of work has demonstrated potential for botanical systems to remediate VOCs, effects associated with static chamber limitations, insufficient chamber volumes, and the sometimes-unrealistic VOC concentrations used bring into question the efficacy of these systems in realistic environments. As such, active botanical biofilters were established concurrently to this body of research, providing greater removal efficiencies than passive systems, and the promising ability to remediate large volumes of air.

The initial studies that incorporated active airflow into biofilter testing observed a relationship between airflow rate, substrate depth and air path porosity on the removal efficiency for various VOCs (Darlington et al. 2000, 2001). With the incorporation of active airflow, increasing pressure drop across the substrate membrane was theorised to increase the rate at which VOCs are able to diffuse into the aqueous phase in the rhizosphere. This development overcame the limitations of a passive pot-plant system as VOCs would need to be passively diffused into the substrate layer (the rate of which is dependent on the chemical’s Henry’s constant), or be translocated through stomatal uptake for remediation to take effect (Guieysse et al. 2008; Irga et al. 2017a, b; Pettit et al. 2017; Wang and Zhang 2011). However, dissolved VOCs may also exit the aqueous phase and return to the ambient air, as determined by their Henry’s constant. To counter this effect, and subsequently increase the remediation potential of botanical biofilters, substrate development was investigated through the addition of effective adsorbents. For example, substantial single pass removal efficiencies (SPREs) for toluene, formaldehyde, benzene and ethyl acetate have been reported for an activated carbon and coconut coir substrate (Aydogan and Montoya 2011; Pettit et al. 2018a; Wang and Zhang 2011). However, the implementation of activated carbon has been seen to effect particulate matter removal efficiencies, and in some cases contribute to ambient PM concentrations (Pettit et al. 2018a). While activated carbon is regarded as the most effective addition for biofilter substrates, research into active biofilter substrate composition is still in its infancy, and a comprehensive study on the capacity to filter a range of pollutants, as well as their effects on plant health and cost practicality is still required.

The phytoremediation of VOCs aside, the implementation of active airflow has drastically increased the remediation potential of these systems for particulate matter (PM). Conventionally, passive systems relied on dry deposition to remove particulate matter, with PM being trapped and stored in the waxy cuticle of leaves, or imbedded into the substrate, given enough time. With active botanical biofiltration, PM can be forcibly embedded into the substrate and root matrix, and sequestered at a significantly higher rate (Lee et al. 2015). Several authors have noted significant PM reductions through the use of active botanical biofilters, where plant selection has been determined to play a significant role. For example, plants with a dense root structure can compress the substrate media, creating a higher pressure drop and more intricate matrix for PM capture and removal from ambient air (Elkamhawy and Jang 2020; Ibrahim et al. 2021; Irga et al. 2017a, b; Pettit et al. 2019a, b).

With the incorporation of airflow comes the concern that the force-aerated moist substrates and biological material could lead to increased indoor humidity, as well as the release of bioparticles (Botzenhart et al. 1984; Engelhart et al. 2009; Hedayati et al. 2004; Soreanu 2016; Staib et al. 1978; Summerbell et al. 1989). Previous studies have observed indoor humidity associated with green walls to be elevated when compared to those without, however the increase in humidity is often to levels deemed comfortable for human habitation (Tudiwer et al. 2017). Several studies worldwide have assessed the release of bioparticles from active green walls, and some elevated particle numbers have been observed, primarily fungal spores. However, no study to date has found elevated concentrations above WHO guidelines, and of the studies that identify the bioparticles, no allergenic or pathogenic species have been detected (Darlington et al. 2000; Fleck et al. 2020; Irga et al. 2017a, b). The *in-situ* assessment of bioparticles is an essential aspect of this technology, as there is the potential for commercial providers to rush or cut corners, especially as the market becomes more popular, potentially leading to changes in substrate physiology, or poor maintenance. This continued monitoring of *in-situ* systems will provide a solid basis for the implementation of active green walls indoors and validate the findings above.

To date, the majority of botanical biofilter research has been conducted on single VOC species, and at concentrations that are often too high to reflect environmental exposure. As commercial interest in this technology grows, it is essential that research is conducted both on single VOCs, as well as mixed VOC sources that are environmentally relevant, such as cigarette smoke, petrol vapour, exhaust emissions etc. Currently, studies have reported significant reductions in environmental tobacco smoke (ETS) in an *in-situ* setting as well as a reduction in traffic associated air pollutants (Morgan et al. 2022; Permana et al. 2022; Pettit et al. 2021; Siswanto et al. 2020). While these early findings validate the technology for the remediation of realistic pollutants, the implementation of active biofilters *in-situ* is still relatively novel. The testing of large commercial systems in a variety of locations globally is still required to assess the potential of this technology to address significant sources of indoor and outdoor air quality contaminants that are largely not treated by conventional technologies.

### Development of active green wall systems

**Fig. 3:**
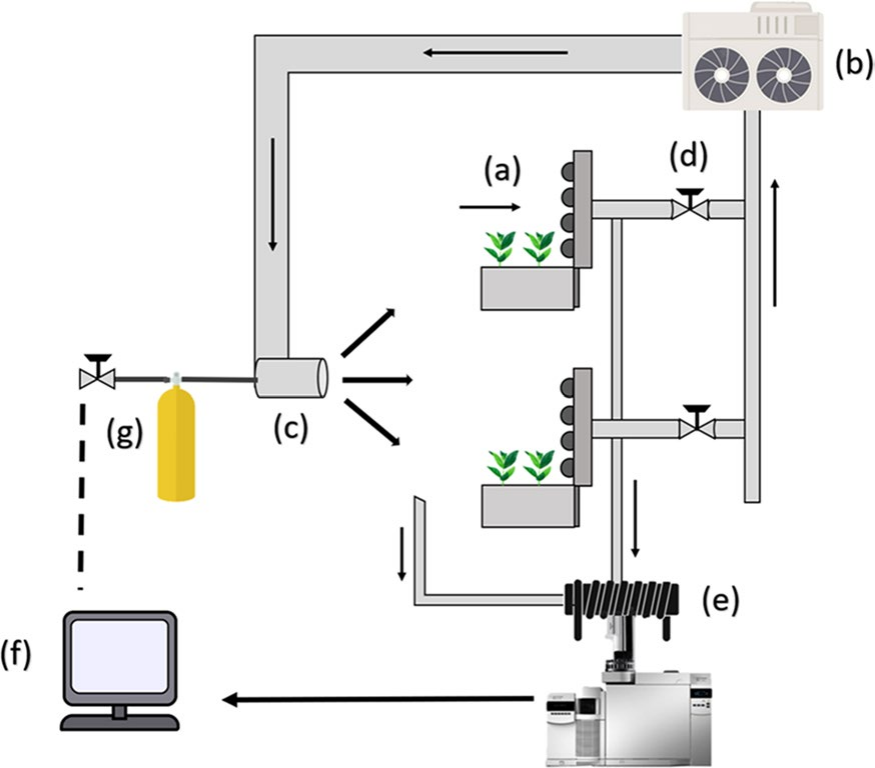
Adapted design of Darlington et al.’s. (2001) green wall bio scrubber study design. Four biofilter modules (only two are shown) were arranged in parallel in terms of air flow in a relatively sealed indoor space. Air was drawn through the biofilters (**a**) by a dedicated air handling system (**b**) and returned to the ambient air mass (**c**). Fluxes through the biofilters were independently controlled with valves (**d**). BTEX levels in the effluent and influent air streams were automatically measured with a gas chromatograph. A solenoid system (**e**) interfaced with the GC selected the sampling site. To control influent VOC concentrations, concentration data were transferred to a peripheral computer (**f**) which activated controlled air flow through one of three specific VOC sources (**g**) (only one shown)

The major limitation of passive potted systems for phytoremediation of indoor spaces is the large number of individual plants required to have a significant, worthwhile effect (Cummings and Waring 2020). Passive systems are limited by the rate at which pollutants diffuse through indoor air and into the plants functional zone, which makes their effects functionally limited in spaces with inadequate air circulation (Soreanu 2016). The original design by Wolverton et al. (1982) (Fig. 1) provided functional improvements in regard to facilitating airflow through the substrate, however it was constrained by a low volumetric capacity (Wolverton et al. 1984). In the early 2000’s, Darlington et al. (2001) solved the issue of adequate planting density (plants per m^2^) through the development of a green wall system with an array of “bio scrubbers” in which ambient air was exposed to the functional areas of the system (Fig. 3). Darlington et al.,’s green wall design integrated plants with a bio scrubber substrate aligned along a vertical plane to considerably increase both the planting density and exposure of the growth substrate to polluted air streams (Gunawardena and Steemers 2019). A porous layer of plant-growth substrate, along with its endogenous microbial community allow active green walls to be effective at the removal of particles and gaseous pollutants simultaneously (Pettit et al. 2019a, b; Torpy et al. 2014). The application of active green wall systems for the phytoremediation of indoor air is a growing industry worldwide, with the demand for these systems being driven by a handful of proof-of-concept studies (Table 4). Active green wall systems development has examined plant selection, airflow rate and substrate composition to determine their effects on increased or sustained pollutant removal. The future prospects for these technologies could be as stand-alone filtration systems, or integrated pre-conditioning systems for HVAC, which may serve to lower the energy consumption of modern buildings (Leavey et al. 2015; Pérez-Urrestarazu et al. 2016).

**Table 4:** A summary of active biofiltration studies that detail the removal of various VOCs from static chambers

Study	Year	Pollutants	Starting concentrations	Plant species	Substrate information	Chamber volume	Removal rate/efficiency	Removal mechanism
(Wang and Zhang)	2011	Formaldehyde and toluene	17 ppb formaldehyde and 2 ppb toluene	*E.aureum*	1.08 m^2^	54,400 L	50.1–98.7%	Activated carbon and porous shale pebbles
(Wang et al.)	2014	Formaldehyde	7.5–10 ppm; and 250 ppm	*E.aureum*	1.08 m^2^	5,100 L	39.5% (biofilter)	Activated carbon pellets and pebbles
(Lee et al.)	2015	PM, formaldehyde, *o* – xylene, xylene, ethylbenzene, toluene, benzene	*Undisclosed*	*D. amoena*	N/A	Undisclosed	40% (PM_10_) 4% (PM_2.5_) 72% (xylene, ethylbenzene, toluene, and TVOC) ≥ 39% (benzene and HCHO)	Soil
(Irga, Paull, et al.)	2017	Particulate matter	~ 700; g.m^−3^ (TSP)	*C. comosum*	0.25 m^2^	216 L	53.5% (TSP) 53.51% (PM_10_) 48.21% (PM_2.5_)	Coconut husk
(Pettit et al.)	2017	PM	19.86 µg/m^3^ (PM_0.3–0.5_) 8.09 µg/m^3^ (PM_5-10_) 142.23 µg/m^3^ (TSP)	*C. orchidastrum, N. glabra, F. lyrata, N.bostoniensis, N.duffii, S.amate, S.arboricola*	0.25 m^2^	216 L	Max removal; 45.78% (PM_0.3 – 0.5_) 92.46% (PM_5-10_)	Coconut husk
(Ibrahim et al.)	2018	PM_2.5_, PM_10_	*Undisclosed*	*E. aureum*	0.14 m^2^	216 L	Removal efficiency: 85% (TSP), 75.2% (PM_2.5_) and 71.9% PM_10_ CADR: 123 L.s^−1^ (TSP), 112.80 L. s^−1^ (PM_2.5_), 107.88 L. s^−1^ (PM_10_)	Kenaf fibre
(Torpy et al.)	2018	Methyl ethyl ketone	30 ppbv	*P.scandens, P.’brazil’ scandens,* *A.antiquum, and S.podophyllum*	1.5 m^2^	30,000 L	56.60%	Inorganic growing media
(Pettit et al.)	2018a	PM, VOCs	~ 500 – 600 ppb (VOCs) N/A (PM)	*N.bostoniensis*	0.25 m^2^	216 L	25.66% higher removal than soil treatment for benzene ~ 78% SPRE (ethyl acetate)	50:50 (coconut husk to granular activated carbon)
(Hung et al.)	2019	CO_2_, formaldehyde, PM_10_	*Undisclosed*	*N. exaltata*	0.38 m^2^	1400 L	Removal capacity: 88.2% (PM_10_), 62.2% (PM_2.5_), 13.9% (CO_2_), 60.4% (formaldehyde)	*Undisclosed*
(Paull et al.)	2019	PM, VOCs, CO_2_	*Undisclosed*	6 Australian native species	0.25 m^2^	216 L	SPRE: 59.04% (Benzene), Australian native plants are less effective for PM and CO_2_ removal, compared to common ornamental indoor plants	Coconut fibre-based substrate
(T Pettit et al.)	2019	PM, VOCs (from lavender oil)	300 ppbv (TVOC) 101.18 µg m^−3^ (TSP)	*N.exaltata, P.obtusifolia, S.arborcola, S.wallisii*	9m^2^	120,200 L	~ 28% over 20 min (TVOC) 42.6% over 20 min (TSP)	Coconut husk
(Thomas Pettit et al.)	2019	Nitrogen dioxide, ozone	6.656 ppm (NO_2_) 7.280 ppm (O_3_)	*S.wallisii* *S.podophyllum*	1.06 cm^2^ 0.901 cm^3^	900 L (flow reactor internal volume)	CADR (m^3^ ·h ^−1^ ·m^−3^ of biofilter substrate) 661.32 and 95.04 (S*.wallisii)* 550 and 23 (*S.podophyllum)* for NO_2_ and O_3_	Coconut husk
(Elkamhawy and Jang)	2020	PM_10_, PM_2.5_	undisclosed	Vegetation (grass or moss), engineering soil and porous material	7 m in height	*In-situ* outdoor	78.5% reduction for PM_2.5_, 47% reduction for PM_10_	Vegetation soil, engineered soil, porous material
(Pettit et al.)	2020	NO_2_, O_3_ and PM_2.5_	5-min ambient averages of 178.6 ppb, 59.4 ppb and 774.7 µg/m^3^ (NO_2_, O_3_, and PM_2.5_	*W.fruticosa,* *M.parvifolium, S.anisophyllus* and *N.domestica*	5 × 20 m^2^	*in-situ* outdoor	SPRE (average); 63.17%, 38.79% and 24.84% (for NO_2_, O_3_, PM_2.5_	Coconut husk
(Siswanto et al.)	2020	Formaldehyde, acetone, benzene and xylene	120–150 ppm (formaldehyde) 127–145 ppm (acetone) 15–35 ppb	*S.trifasciata* *C.comosum*	500 cm^2^	24 m^3^	80–90% (TVOC)	1:1 mix of soil (40:50% clay, 25–30% silt and 25–30% sand) and coconut coir
(Ibrahim et al.)	2021	PM_2.5_, PM_10_ and TVOC	~ 18–25 mg.m^−3^	*E.aureum*	0.05 m^3^	240 L	Removal efficiencies: 54.5 ± 6.04% (PM_2.5_) 65.42 ± 9.27% (PM_10_) 46 ± 4.02% (VOC)	Kenaf fibre
(Suárez-Cáceres et al.)	2021	TVOC and n- hexane	5.69–7.51 mg.m^−3^	*N.exaltata L*	2 X 0.18 m^2^	128 L	Reduction rate: 0.17 and 0.1 mg.m^−3^ h	Mixture of coconut fibre and peat
(Pettit et al.)	2021	NO_2_, O_3_ and PM_2.5_	N/A	*M.parvifolium, S.anisophyllus* and *N.domestica*	5 × 20 m^2^	*in-situ* outdoor	SPRE (average); 71.5%, 28.1%, 22.1% (NO_2_, O_3_, PM_2.5_)	Coconut husk
(Abedi et al.)	2022	Formaldehyde	0.3 – 2 ppm	*Epipremnum aureum (4 per modules), Syngonium* *podophyllum (4 per modules), Chlorophytum comosum (4 per modules),* *Peperomia obtusifolia (2 per modules), Pilea cadierei (1 per modules), and* *Aglaonema treubii (1 per modules)*	0.25 m^2^	50 L	SPRE range 47.05—99.99% in all systems CADR 17.6 m^3^/h	Granular activated carbon, leca and commercial pot soil
(Morgan et al.)	2022	Environmental tobacco smoke (ETS), all size fractions of PM	Full cigarette over 8 min (35 mL puff volume with 1 puff per min)	*S.wallisii*	0.25 m^2^	216 L	SPRE; 43.26% TVOC 34.37% TSP	Coconut husk
(Permana et al.)	2022	PM_1_, PM_2.5_, PM_10_, formaldehyde and acetone from tobacco smoke	2.9–3.0 mg.m^−3^ (PM_1_ and PM_2.5_) 3.6–3.7 mg.m^−3^ (PM_10_) 123–148 mg.m^−3^ (Formaldehyde) 9.5–12 mg.m^−3^ (acetone)	*S. trifasciata*	0.05 cm^3^	24,000 L (Testing room)	Removal of PM_x_ over 8 h; 140–250 µg m^−3^ (PM_1_) 147–257 µg m^−3^ (PM_2.5_) 212–455 µg m^−3^ (PM_10_) Removal efficiency over 24 h; 45–69% (formaldehyde) 31–61% (acetone) 40–65% (TVOC)	1:1 mix of soil and coconut coir

Table includes pollutant type, starting concentration, plant species used, size of the active botanical biofilter, room or chamber volume study was conducted in, the efficiency of the system for each pollutant and substrate information

*TVOC* total volatile organic compounds; *CADR* clean air delivery rate; *SPRE* single pass removal efficiency

### Effect of airflow pollutant removal

The key component of active green wall systems is the consistent supply of airflow through the substrate layers which facilitates sustained filtration (Pettit et al. 2019a, b). The supplied airflow to an active system directly effects the pollutant gas residence time within the substrate matrix, also known as bed residence time. Bed residence time governs the efficacy of VOC removal by active green walls, as gaseous pollutants must transfer from the gas to the liquid phase before they can be made available for rhizospheric degradation (Darlington et al. 2001; Delhoménie and Heitz 2003; Halecky et al. 2016). As such, active mechanically induced airflow within active green wall systems allows pollutant removal to be reported as either a single pass removal efficiency (SPRE) or as a clean air delivery rate (CADR); which are metrics also used for assessing the performance of conventional air handling systems.

SPRE is the proportion of a target pollutant that is filtered by the biofilter during each pass through a filtration system. The SPRE can be calculated using Eq. (1).

Single pass removal efficiency calculation.

1 $$\left( {SPRE = \left( {\frac{{{\rm{inlet}}\;{\text{pollutant}}\,{\rm{concentration}} - {\rm{Outlet}}\;{\rm{pollutant}}\;{\rm{concentration}}}}{{{\rm{Inlet}}\;{\rm{pollutant}}\;{\rm{concentration}}}}} \right) \times 100} \right)$$

One advantage of biofiltration is the non-specific removal capacity of the systems, unlike many mechanical solutions. Pollutants can be removed simultaneously, and therefore SPREs can be calculated for each. Removal rates for single chemicals can be expressed as CADR if the airflow rate is known (Eq. (2)).

Clean air delivery rate calculation.

2 $$CADR = SPRE \times Biofilter\;airflow\;rate$$

This subsequent CADR is specific to each pollutant, as SPREs only refer to the reduction of single pollutants. The use of CADR allows for comparison against other systems. When calculations are made for indoor systems, the CADR is taken as a function of the room volume to calculate the biofilter refreshment capacity (Eq. (3)). Combining BRCs for pollutants relative to an application could be used to estimate the air exchange rate of a system.

Biofilter refreshment capacity

3 $$Biofilter\;refreshment\;capacity(BRC) = \frac{{CADR}}{{Volume\;of\;room}}$$

These above equations can be incorporated into biofilter design to establish the required biofilter dimensions required to clean a room of a given size and to provide a necessary air exchange rate (Eq. (4)).

Required biofilter volume

4 $$Biofilter\;volume = \frac{{BRC \times gas\;residence\;time \times Volume\;of\;room}}{{SPRE}}$$

**Fig. 4:**
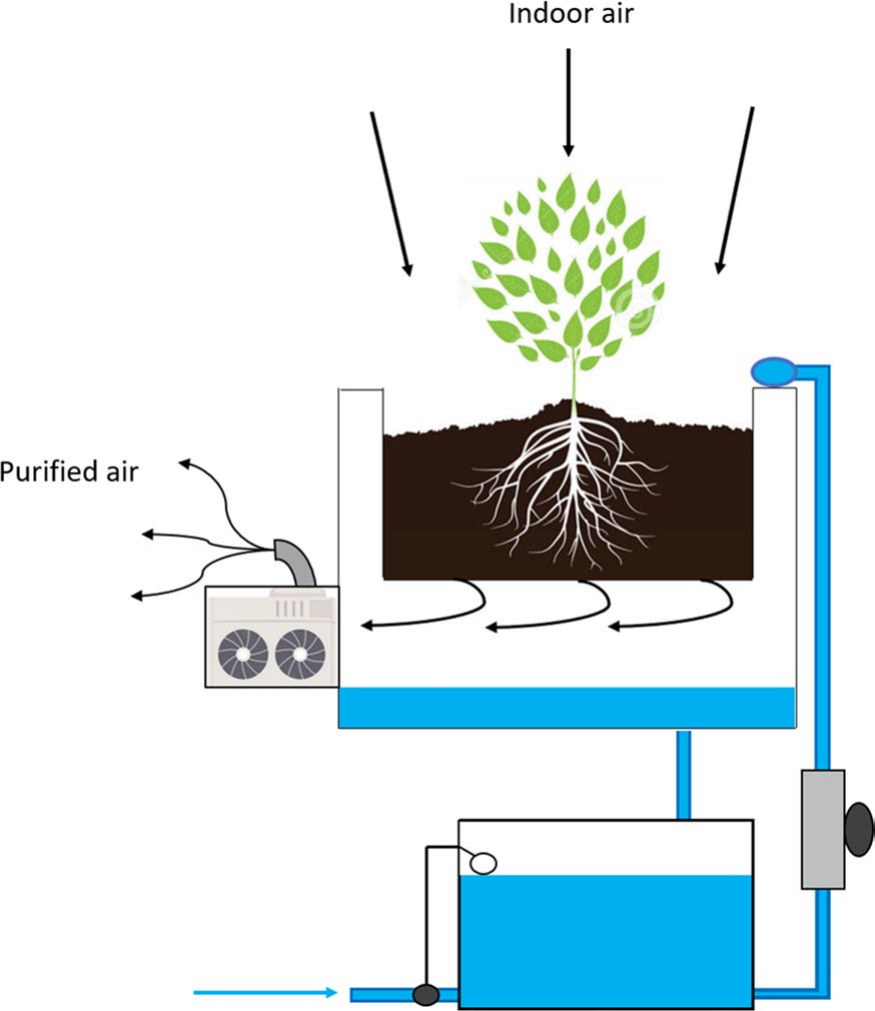
Active botanical air filter. Airflow is directed through a horizontal planted surface and downwards through activated carbon and shale pebble substrate. Image adapted from Wang and Zhang (2011)

If an active green wall system is to achieve increased and sustained indoor pollutant removal, it is essential to assess the physiochemical factors that affect airflow in green walls in their development. The study of airflow through plant growth substrate is a relatively under studied field of research. While Darlington et al. (2001), Delhoménie et al. (2003), determined that VOC removal rates were highest with slower airflow rates, the highest CADRs were achieved with higher airflow rates. The authors (Darlington et al. 2001; Delhoménie et al. 2003) proposed that diffusion of VOCs through the aqueous phase acted as a rate limiting step, suggesting that increasing airflow rates through systems will further increase their efficiency. However, it is likely that the optimal airflow rate to achieve the greatest CADR is pollutant dependent (Llewellyn et al. 2002), with active systems creating a pressure drop due to the air resistance generated by the substrate membrane, which dictates a VOCs ability to enter and exit the aqueous phase determined by their individual Henry’s Constants (Guieysse et al. 2008; Pettit et al. 2017; Wang and Zhang 2011). Wang and Zhang (2011) found higher removal rates for formaldehyde than toluene when the water content in their substrate membrane was higher and airflow was directed downwards and through the substrate depth (Fig. 4), likely due to the hydrophobic nature of toluene preventing it from rapidly moving into the aqueous phase. It was been suggested that due to the inviolable requirement for an irrigated substrate in active green walls, hydrophilic compounds will be remediated with greater efficiency at higher airflow rates, while hydrophobic compounds will require greater residence time within the biofilter media to allow them to solubilise in the aqueous phase (Guieysse et al. 2008; Pettit et al. 2018a). Understanding the effect airflow has on the pollutant cleaning rates of these systems is important in order to further develop the technology, as identification of optimal air speeds to achieve efficient removal rates of specific pollutants will allow other system characteristics such as the botanical components to be chosen to best meet the required airflow needs for specific green wall systems.

### Effects of plant selection

Research into how the physical structure of plants effects airflow through active system is relatively novel, however a correlation between airflow rate and plant efficiency to reduce pollutant concentrations has been established (Delhoménie et al. 2003). Currently this property is under addressed in the literature and is an important element in the optimisation of active green wall systems, as plant species selection appears to directly influence airflow and pressure drop across the substrate, thus effecting bed-residence time and CADRs (Irga et al. 2018). Irga et al. (2017a, b) assessed the atmospheric particle removal efficiency of an in-room biofilter system, finding that root structure and thus plant species may affect the air-filled porosity of the substrate media. Comparatively, Pettit et al. (2017) demonstrated that varying plant species and their respective root systems affect pressure drop across the filter media, with those systems showing higher pressure drop correlating with higher PM removal. It was suggested that the dense root structure compresses the substrate resulting in increased pressure drop across the substrate and resulting in higher filtration efficiency. Whilst these effects have been demonstrated for PM, there is little research indicating the effect of pressure drop and airflow rate on many pollutants, including NO_x_, SO_x,_ CO and many VOCs.

### Effects of substrate physiology

Substrate composition and physical properties are important factors in botanical biofiltration not only because of its need to sustain plant life, but also because factors like porosity, surface area, compaction, water holding capacity, and adsorption capacity are key determinants in a system’s pollutant filtration capacity (Irga et al. 2018). A wet substrate is clearly important to sustain plant health, however the presence of water has been shown to amalgamate soil particles, creating a substrate that is highly porous, thus allowing larger volumes of air to pass through, leading to less residence time within the substrate and reduced SPRE (Abdo et al. 2016). It has also been shown that different substrates also influence botanical biofilter performance. The best performing substrates articulated within the literature are those that incorporate granular activated carbon (GAC) due to its high surface area containing many hydrophilic and hydrophobic adsorption sites for pollutants (Pettit et al. 2018a). It has been proposed that GAC can improve the removal of hydrophobic contaminants such as benzene by trapping water molecules in its hydrophilic regions, creating a larger driving force for the entry of hydrophobic pollutants into the aqueous phase (Wang 2011). Wang and Zhang (2011) found high removal rates for formaldehyde while utilising a substrate matrix consisting of 1:1 mix (by volume) of GAC to shale pebbles. Pettit et al. (2018a) replicated this composition, demonstrating higher removal rates for benzene and ethyl acetate than a solely coconut based substrate. Aydogan and Montoya (2011) tested multiple growing media under varied watering conditions for the removal of formaldehyde within a sealed chamber, and determined that substrates containing activated carbon performed best under all conditions.

However, while the substitution of plant growing media for GAC can contribute to higher removal efficiencies for some contaminants, GAC is also efficient in removing botanically important nutrients such as nitrates, ammonium and phosphates (Khalil et al. 2017; Zanella et al. 2015; Zhu et al. 2016), leading to a potential decline in plant health should these nutrients become limiting. As such, GAC must be used in conjunction with another substrate material, and in in concentrations that will not harm plant viability. In the studies mentioned previously, Wang and Zhang et al.’s. (2011) 1:1 mix of GAC and shale pebbles demonstrated an ability to support plant growth for 300 days with effective pollutant removal efficacies. Comparatively, Pettit et al’s (2018a) 1:1 mix of GAC and coarse coconut coir successively supported plant life for over 280 days. These findings indicate that 50% or more GAC: plant growth substrate should be acceptable to maintain plant health in most cases, although further work comparing substrate interactions with activated carbon or other absorbents within botanical biofilters is needed. For the commercial adoption of a composite substrate material to be viable, it first must be experimentally addressed with a range of plant species and pollutant types. This will further facilitate the optimisation of active green walls for the management of indoor air quality.

In regards to PM removal, unlike potted plants where removal is based solely on deposition on the plant foliage, active systems pull air through a growth medium having many of the same properties as conventional filters. Removal efficiencies for PM in previous studies tend to increase as PM particle size increases (Irga et al. 2017a, b; Kim et al. 2021; Pettit et al. 2017). Pettit et al. (2017) found that PM SPRE could be enhanced with appropriate plant species selection, whereby plant species with denser, more complex root systems create a more compact substrate with altered pressure drop properties that positively influence PM removal efficiency. Considering this, Pettit et al. (2019a, b) noted considerable PM mitigation by an *in-situ* active green wall within a Beijing classroom containing a range of common indoor species, with the active system comparatively outperforming filter within the HVAC system of the classroom for PM removal at all size classes. It is possible that alteration of other substrate properties which influence pressure drop, as well as other physiochemical characteristics of a system’s substrate media could affect the subsequent PM removal performances, but further work in this area will be required to elucidate these effects.

### Plant microbe interaction

While additions to substrate media can provide additional adsorption sites for various pollutants, other biotic factors must be considered when optimising substrate media. Microbial communities within the rhizosphere of botanical biofilters are largely responsible for the removal efficiencies of the system when it comes to VOC removal. Therefore, it is imperative that substrate optimisation also considers the rhizospheric bacterial community. Irga et al. (2013), found that benzene removal in hydroculture substrates was slower than traditional potting mixes, relating this too the more diverse bacterial community within soil based substrate media resulting in more effective rhizospheric pollutant degradation. Limited research into optimising botanical biofilter systems through bioaugmenting or biostimulating the substrate with specific VOC degrading bacteria has been performed. Torpy et al. (2013) compared benzene removal between ordinary potted plants and plants with a substrate wherein the benzene degrading microbial community was specifically biostimulated, finding the system with enhanced bacterial community to have higher benzene removal. Likewise, Sriprapat and Thiravetyan (2016) demonstrated that inoculation of leaf surfaces with endophytic benzene-degrading bacteria showed an increase benzene removal efficiency compared to ordinary potted plants. This has also been seen to occur when inoculating leaf surfaces with cultures of toluene-degrading bacteria (De Kempeneer et al. 2004). It should be noted these studies involved passive systems exposed to high concentrations of VOCs; it remains unknown whether inoculated endophytic and phyllospheric bacterial communities could be sustained within *in-situ* active systems.

### Air supply effects

**Fig. 5:**
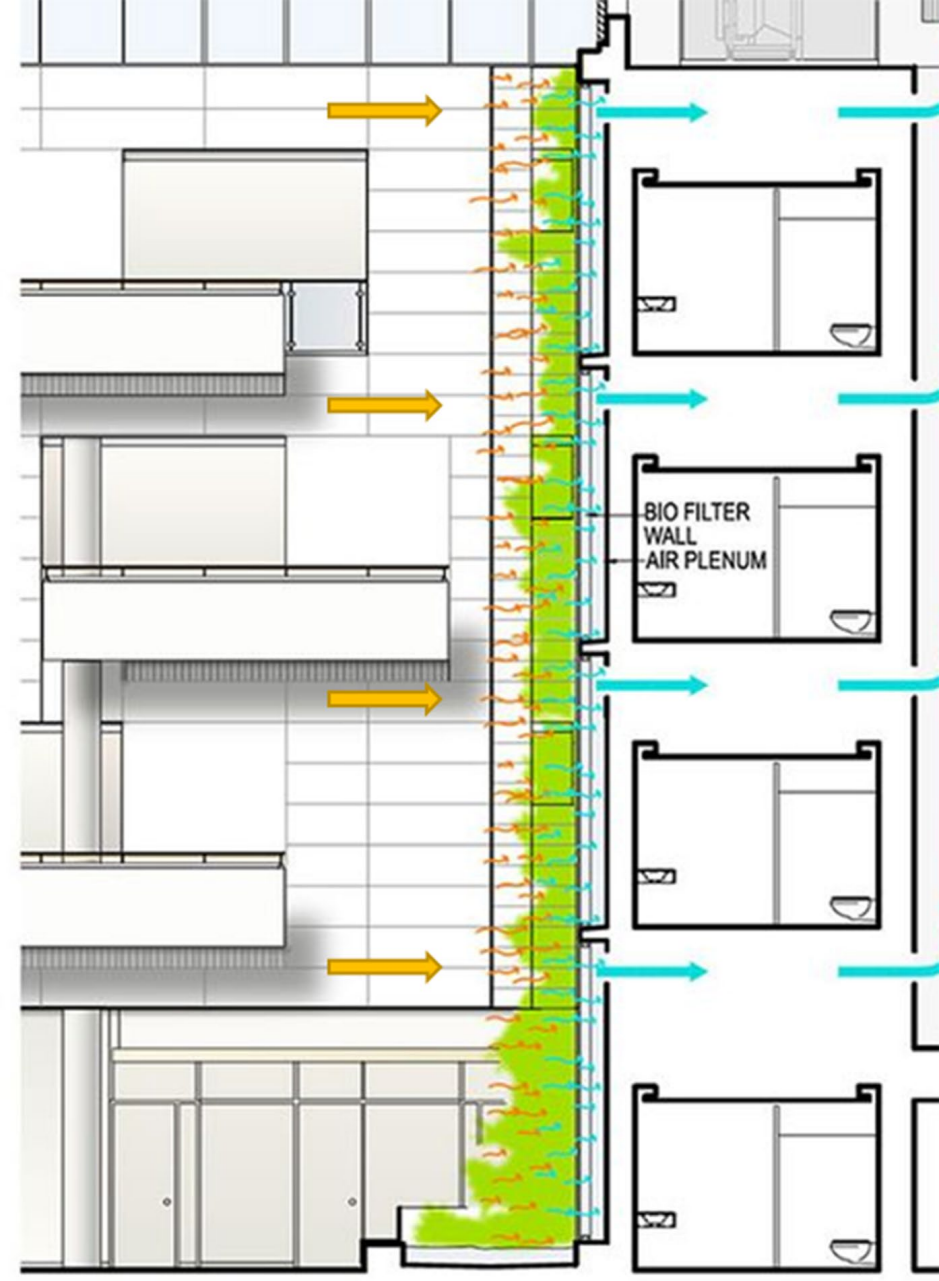
Architectural concept drawing of green wall connected to building’s HVAC system, drawing polluted air (orange arrows) through the green wall and then sending the filtered ‘clean’ air (blue arrows) out to the occupied spaces through the supply air system. Image from Stoughton (Stoughton 2015)

Active green walls have been proposed to be alternatives or additions to conventional HVAC systems. It has been conceived that active biofilters could be incorporated into the HVAC conditioning system to increase both the performance and filter longevity of the HVAC such as the concept shown in Fig. 5 (Wang and Zhang 2011). Conventional HVAC relies on the mechanical filtration of pollutants, requiring regular mechanical system maintenance and the replacement of filter materials. Without the required maintenance or in instances with heavy pollutant exposure, the filter materials clog, resulting in an increase in pressure drop, and therefore an increase in energy consumption, a reduction in performance, air distribution efficiency and system capacity (Nassif 2012). Typical reverse cycle air conditioning systems in buildings have an energy expenditure of USD $0.54 per hour for a medium sized 36m^2^ room. In large areas (50m^2^), HVAC systems typically costs between $0.70 and $0.95 per hour (O'Niel 2019). A study conducted by Wang and Zhang (2011) demonstrated the potential of botanical biofilters to be integrated within HVAC systems, both increasing filtration performance and providing the ability to remediate VOCs and CO_2_ without a reliance on flushing with outdoor air. Cost assessments of these systems are in their infancy, however there are several proof-of-concept studies. Nelson and Bohn (2011) assessed the cost of soil biofiltration in comparison to other methods of air purification and confirmed that the use of botanical filtration offers a cost reduction to users (Fig. 5).

### Active green walls for urban air quality management

Traditional urban forestry such as trees, hedges and shrubs has been thoroughly researched for its ability to remove urban pollutants (Abhijith and Kumar 2019), with an estimated annual removal of ~ 711,000 t urban pollutants (consisting of PM_10_, NO_2_, O_3_, SO_2_, CO) within the United States, providing a service valued at ~ $3.8 billion (Nowak et al. 2006). However there have been instances where high density vegetation areas such as heavy tree canopies have been shown to restrict the diffusion of air pollution from traffic emissions, causing a localised increase in the concentration of ground level air pollution (Gromke et al. 2008).

**Fig. 6:**
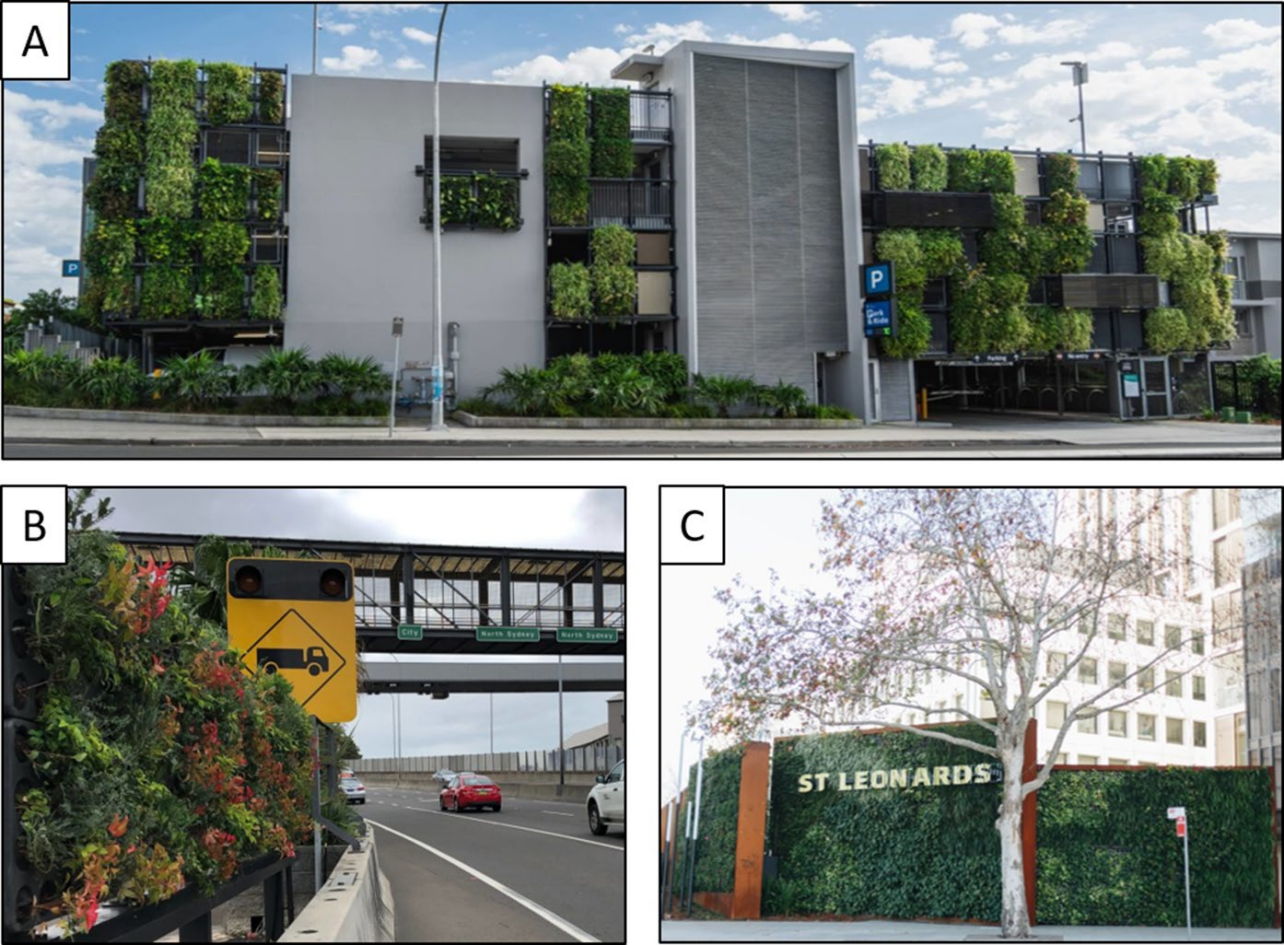
Large scale active green walls in Sydney, Australia. **A** Manly vale B-line carpark. **B** Eastern distributor motorway, Sydney. **C** Mitchell Street Plaza, St Leonards (Junglefy 2023)

Existing systems for urban air pollution mitigation such as vegetation barriers and solid roadside barriers primarily work through pollution dispersion rather than the reduction of ambient pollutants (Gallagher et al. 2015; Tong et al. 2016). Some cities are developing massive air filtering devices, an example being the large air cleaner installed in Xian, central China to treat up to a 10 square-kilometre area (Nedjati et al. 2022). Given the scalability of modular active green wall systems, they would be viable alternatives that serve more functions that just to purify air. To date, active green wall research has been predominately limited to indoor air quality investigations and laboratory studies, however nascent manipulative experiments for the proof of concept for active systems to be used as an outdoor air filtration system have been performed (Thomas Pettit et al. 2019a, b; Pettit et al. 2020, 2021). Unlike vegetation and solid roadside barriers that shift pollutant dispersion (Pettit et al. 2021), these studies demonstrate that the application of airflow in botanical biofiltration could be used to effectively remove air pollutants from ambient air. With the primary restriction on practicality being the large size of the systems that would be required. It is suggested that future research test the performance of the targeted placement of active green systems in areas where air pollution can be directly filtered at its source, for example, car parking stations and traffic tunnels (Fig. 6).

### *Removal of NO*_*2*_* and O*_*3*_

Plants have demonstrated an ability to uptake atmospheric NO_2_ and incorporate it into their nitrogen pathways (Vallano and Sparks 2007). Microbiota within the plant rhizosphere utilize nitrogenase enzymes to fix NO_2_ and break it down into ammonia, which the plant can use to produce biomolecules to support plant growth (Weyens et al. 2015). However it is likely that uptake through the plant’s stomata remains the primary method of NO_2_ removal (Geßler et al. 2002). NO_2_ may also be accumulated in plants in the form of nitrate and nitrite, subsequently being reduced by nitrate and nitrite reductases, generating NH4, which is then assimilated to glutamate through the GS-GOGAT pathway (Lee et al. 2021; Singh and Verma 2007; Wei et al. 2017).

Ozone is adsorbed by deposition on the cuticle of the plant and absorption through stomatal apertures (Fares et al. 2010). As cuticle deposition is only effective if there is high surface moisture on the plant, absorption into the stomata is considered the main contributor to ozone uptake (Altimir et al. 2006; Loreto and Fares 2007). During gas phase transfer, ozone reacts with waxes, salt ions and biogenic VOCs on the cuticle (Fares et al. 2010). The effects stomatal ozone absorption has on plants is not fully understood, however it has been hypothesised that once entering the stomata, it reacts with compounds in the apoplast to form reactive oxygen species (Oksanen et al. 2004). Due to the ability of plants to take up both NO_x_ and O_3_ from the atmosphere, active botanical biofilters are regarded as a possible solution to this aspect of the urban air quality crisis (Pettit et al. 2021).

Proof-of-concept evidence for NOx removal has been produced by Pettit et al. (2019a, b), using replicate active green walls within a closed loop flow through experiment, which observed exponential decay of high concentrations of NO_x_ and NO_2_ for two different plant species (*S.wallisii* and *S.podophyllum*). Nevertheless, longer-term experiments under *in-situ* conditions are needed to establish practical removal rates and plant health exposure to air pollution. The existing examples of such studies are discussed in Sect. 4.2.

### Limited in-situ testing

The small ground and canopy footprint of green walls allows these systems to be incorporated in spatially constrained urban environments (Abhijith et al. 2017). Currently there is some indication that active green walls can be used in the urban environment to provide additional benefits besides urban pollutant abatement, however research has been limited by the paucity of available *in-situ* active systems to study. Active green walls have demonstrated some ability to filter urban stormwater, to be used as acoustic buffer and to enhance the biophilic design of urban spaces, which is documented to have substantial psychological impacts on urban dwellers (Haviland-Jones et al. 2005; Lohr et al. 1996; Ulrich 1979). These benefits alone demonstrate the inherent value of green infrastructure as a sustainable urban technology (Fleck et al. 2022; Pettit et al. 2021). Although research on outdoor *in-situ* testing is in its infancy, large effect sizes demonstrated for indoor air pollutant removal indicate that this technology should be considered of potential value for the remediation of outdoor environments. Pettit et al. (2020), examined the ability of an active green wall to reduce elevated ambient levels of NO_2_, O_3_ and PM_2.5_ during the 2019 Sydney, Australia wildfires, during which concentrations for NO_2_, O_3_ and PM_2.5_ were observed at 100, 76 and 127 times greater than the normal average range across a 14-day sampling period. Over the sampling period, average SPREs of 63.17%, 38.79% and 24.84% for NO_2_, O_3_ and PM_2.5_ respectively were recorded for two 5m^2^ green walls tested *in-situ*. This was further expanded on in Pettit et al. (2021), who implemented three different active green wall designs for the filtration of NO_2_, O_3_ and PM_2.5_ from roadside ambient air in Sydney, Australia. At each site, ambient concentrations of all pollutants detected within the effluent airstreams of the green walls were lower than ambient concentrations, with average SPREs of 71.5%, 28.1% and 22.1% for NO_2_, O_3_ and PM_2.5_ respectively. These initial field assessments demonstrate the potential for this technology to be implemented as an effective urban pollutant mitigator. In light of these preliminary studies, several infrastructure-scale systems are planned for installation in critical locations around Australia, where future work should aim to assess the influence of these systems on the general ambient air quality conditions experienced by populations residing in proximity to these systems.

### Future directions

Active botanical biofiltration is a rapidly growing technology showing potential for the phytoremediation of both indoor and outdoor air pollutants. There is evidence suggesting that botanical biofilters could maintain indoor air quality through the recirculation of indoor air as standalone, or as HVAC integrated solutions. However, performance developments are needed to reach this goal, with a substantial body of work required to test the performance of these concepts. While there is limited research relating to the CADR of active biofilters, the CADR achieved by Wang and Zhang (2011) provides a promising insight into the potential for an integrated biofilter/HVAC system. Discouragingly, the current literature utilises a range of experimental approaches to evaluate the performance of active systems, leading to inconsistent results amongst a range of biofilter designs. With the use of different pollutants, wall sizes, time frames and pollutant concentrations, valid comparisons between systems is difficult. To address this, a standardised approach to the reporting of pollutants/concentrations, wall properties (size/volume/plant area) should be established and metrics such as pressure drop and airflow should be reported for all future studies to ensure valid comparisons can be made across systems.

While there is ample literature that has evaluated the capabilities of various plant species to remove pollutants, comparatively, there is substantially less quantitative evidence to support the use of biofiltration systems long term for the remediation of air pollution (Paull et al 2018), if botanical biofilters are to be used in highly polluted environments the plants used in these systems must be resilient. While Paull et al. (2018) concluded most green wall plant species have the capacity to withstand high pollutant environments long term experimentation that assesses multiple plant species and pollutants could provide insight into possible maintenance needs and evaluate the associated costs of plant health maintenance which would assist in higher public acceptance for the technology (Lee et al. 2021). Additionally, the long-term exposure to environmental pollutants has an unknown effect on the rhizospheric microbial community, as well as its function under short term exposure to very high concentrations of pollutants. Future work that places an emphasis on microbial community response to mixed and varied concentrations of pollutants would be of substantial benefit to the field. Exact profiling and understanding of metabolic pathways, genes and enzymes involved in microbial remediation within active green walls is a new area of study and could allow improved screening of plant species to advance pollutant removal efficiency of these systems (Khalifa et al. 2022).

In addition to this, indoor systems are likely to influence indoor CO_2_ concentrations through photosynthesis and respiration, it is possible that plants with high VOC removal rates grown under low light conditions may emit CO_2_ as an end product of degradation. As such it is critical that *in-situ* systems be assessed for their potential to reduce reliance on HVAC and the associated energy savings, as well as the effect of abiotic factors substrate moisture, composition, along with plant species screening or the use of C3 and CAM plants species to limit any CO_2_ emissions, combination of such a system with adequate light levels of 250 μmol m^−2^ s^−1^ or greater (Torpy et al. 2017) would have the potential to efficiently remove substantial CO_2_ within indoor environments (Treesubsuntorn and Thiravetyan 2018). Future comprehensive indoor trials should thus not only evaluate the pollutant removal potential of botanical biofilters, but also quantitatively evaluate their ability for holistic environmental quality management for the indoor environment. Alongside this, as botanical biofilter technologies continue to be presented as a sustainable and low energy solution to maintain habitable indoor air quality and as active ambient air quality remediation solutions, comparative energy assessments with conventional air quality management technologies would contribute significantly to the current body of work within this research field.

Since the 2019 outbreak of the COVID 19 pandemic, ambient air pollution has reduced worldwide. However, people are also spending increased amounts of time indoors, placing increased risk of exposure to accumulated human derived VOCs. Hundreds of VOCs are emitted from the human body from the breath, blood and skin (Shirasu and Touhara 2011) with exposure causing discomfort and contributing to illnesses. Characterising the removal potential for these VOCs as well as the potential of biofiltration systems to remove COVID and other viruses from the airstream would be significant contributions to this field of research.

## Summary

Since the recognition of potted plants for improving indoor air quality, research has progressed past the capabilities of simple passive plant systems to the development of active botanical biofilters. The existing literature has demonstrated the potential of this technology, which is reflected by the growing adoption of active botanical biofiltration as a commercial solution to both indoor and outdoor air pollution, presenting opportunities to combine this technology with already established mechanical systems like HVAC to reduce energy use. Along their small ground and canopy footprint makes active botanical systems an attractive technology for air quality enhancement and their low cost compared to conventional air filtration devices gives them major value to sustainable urban design both indoors and outdoors. While there are many promising findings to date and consistent industry growth, further research is needed before this technology will become widely adopted and implemented within indoor and outdoor environments. To further validate the potential of these systems for air quality remediation, reproducible laboratory and field experimentation is required to quantify the effects and variances with respect to system designs, as-well as the influence this has on airflow and its overall effects on biofilter performance. Continued contributions within this field, especially in relation to *in-situ* studies on outdoor active green wall systems, will act as a means to increase public awareness serving to further promote these systems as a priority means within sustainable building practices for the reduction of human health impacts from air pollution.
